# Expression of type one cannabinoid receptor in different subpopulation of kisspeptin neurons and kisspeptin afferents to GnRH neurons in female mice

**DOI:** 10.1007/s00429-021-02339-z

**Published:** 2021-07-14

**Authors:** Tamás Wilheim, Krisztina Nagy, Mahendravarman Mohanraj, Kamil Ziarniak, Masahiko Watanabe, Joanna Sliwowska, Imre Kalló

**Affiliations:** 1 Laboratory of Endocrine Neurobiology, Institute of Experimental Medicine, P.O. Box 67, Budapest, 1450 Hungary; 2 Laboratory of Neurobiology, Department of Zoology, Poznan University of Life Sciences, Poznan, Poland; 3 Department of Anatomy, Hokkaido University School of Medicine, Sapporo, 060-8638 Japan; 4 Department of Neuroscience, Faculty of Information Technology, Pázmány Péter Catholic University, Budapest, Hungary; 5 Doctoral School of Neurosciences “János Szentágothai”, Semmelweis University, Budapest, Hungary

**Keywords:** Endocannabinoid, Retrograde signaling, Kisspeptin, GnRH, Mouse, In situ hybridization

## Abstract

The endocannabinoids have been shown to target the afferents of hypothalamic neurons via cannabinoid 1 receptor (CB1) and thereby to influence their excitability at various physiological and/or pathological processes. Kisspeptin (KP) neurons form afferents of multiple neuroendocrine cells and influence their activity via signaling through a variation of co-expressed classical neurotransmitters and neuropeptides. The differential potency of endocannabinoids to influence the release of classical transmitters or neuropeptides, and the ovarian cycle-dependent functioning of the endocannabinoid signaling in the gonadotropin-releasing hormone (GnRH) neurons initiated us to study whether (a) the different subpopulations of KP neurons express CB1 mRNAs, (b) the expression is influenced by estrogen, and (c) CB1-immunoreactivity is present in the KP afferents to GnRH neurons. The aim of the study was to investigate the site- and cell-specific expression of CB1 in female mice using multiple labeling in situ hybridization and immunofluorescent histochemical techniques. The results support that CB1 mRNAs are expressed by both the GABAergic and glutamatergic subpopulations of KP neurons, the receptor protein is detectable in two-thirds of the KP afferents to GnRH neurons, and the expression of CB1 mRNA shows an estrogen-dependency. The applied estrogen-treatment, known to induce proestrus, reduced the level of CB1 transcripts in the rostral periventricular area of the third ventricle and arcuate nucleus, and differently influenced its co-localization with vesicular GABA transporter or vesicular glutamate transporter-2 in KP neurons. This indicates a gonadal cycle-dependent role of endocannabinoid signaling in the neuronal circuits involving KP neurons.

## Introduction

Evidence increases for a broad involvement of the endocannabinoid signaling employed by preoptic/hypothalamic neurons/e.g., for corticotropin-releasing hormone (CRH), thyrotropin-releasing hormone (TRH), oxytocin (OT), and vasopressin (VP)—see (Di et al. 2003); for proopiomelanocortin (POMC)—see (Hentges et al. 2005); for gonadotropin-releasing hormone (GnRH)—see (Farkas et al. 2010) to regulate the activity of their afferents and thereby to influence their own excitability at various physiological and/or pathological processes. Interfering with this endocannabinoid signaling, i.e., blocking of 2-arachidonoylglycerol (2A-G) synthesis or antagonizing CB1-mediated actions prevent the endocannabinoid-mediated inhibition of GABA release from the afferents, as it was reported for, e.g., GnRH (Farkas et al. 2010) and POMC neurons (Hentges et al. 2005). Very few data are available, however, about the physiological/pathophysiological conditions and target system of an enhanced endocannabinoid signaling, which may recruit other than the GABAergic afferents. This could be similar to the outcome of treatments with CB1 agonists resulting in a reduction both the inhibitory and excitatory postsynaptic currents (IPSCs and EPSCs) in target neurons (Hentges et al. 2005).

Kisspeptin (KP)-producing neurons are known to form afferents of multiple neuroendocrine cells (Clarkson and Herbison 2006). They are located in three major sites, i.e., the preoptic area (POA), the arcuate nucleus (ARC), and the medial amygdaloid nucleus (ME) (Clarkson et al. 2009; Lehman et al. 2013), where they establish local connections (Comninos et al. 2016; Stephens and Kauffman 2017; Krajewski et al. 2010; Qiu et al. 2018), as well as project to distant target areas including the POA (Qiu et al. 2018), supraoptic (SON), and paraventricular (PVH) nuclei of the hypothalamus (Yeo et al. 2016). Their processes play a key role in mediating the positive and negative estrogen feedback to GnRH neurons (Ohkura et al. 2009), which is based on their direct genomic and non-genomic (Mittelman–Smith et al. 2012) responses to estrogen, and direct connections to GnRH neurons. They are also implicated in conveying circadian (Vida et al. 2010; Williams et al. 2011), metabolic (Clarke and Arbabi 2016; Wahab et al. 2013), and limbic (Comninos et al. 2016; Stephens and Kauffman 2017) signals to GnRH neurons. In addition, they provide a rich innervation of OT (Seymour et al. 2017; Liu and Herbison 2016; Scott and Brown 2013), VP neurons (Liu and Herbison 2016) in the SON and PVH, and POMC (Higo et al. 2017; Qiu et al. 2018), and tyrosine hydroxylase (TH) neurons (Sawai et al. 2012) in the ARC, and the functional significance of these neuronal connections, however, is incompletely understood.

Besides producing various neuropeptides (neurokinin B, dynorphin, galanin) (Goodman et al. 2007; Murakawa et al. 2016; Kallo et al. 2012; Porteous et al. 2011) or biogenic amines (dopamine) (Clarkson and Herbison 2011; Skrapits et al. 2015; Bardoczi et al. 2018), subpopulations of kisspeptin neurons use classical neurotransmitters like glutamate and GABA, which contribute to the excitatory and/or inhibitory innervation of the neuroendocrine cells. Contrasting the peptide-type transmitters, these classical neurotransmitters are released spontaneously, as well as at an evoked manner, which raises a potential role of endocannabinoid signaling to discretely influence the release of neurotransmitters/neuromodulators from the different kisspeptin subpopulations. The regulation could happen with different efficacy in the GABAegic and glutamatergic subpopulations of KP neurons, similarly to those observed for the G-protein-dependent signaling of CB1 in cortical principal versus interneurons (Steindel et al. 2013).

Electrophysiological recordings indicate ovarian cycle-dependent alterations in the GABAergic and glutamatergic inputs to GnRH neurons (Balint et al. 2016; Farkas et al. 2018). It was also reported that the endocannabinoid signaling operates in a phase-dependent manner; in metestrus, endocannabinoids suppress the postsynaptic currents (PSCs) in GnRH neurons, whereas in proestrus, they do not seem to contribute to the increase of these events (Balint et al. 2016; Farkas et al. 2018). By establishing many critically important estrogen-sensitive pathways, it is of interest whether (a) GABAergic and/or glutamatergic subpopulations of KP neurons express CB1 mRNAs, (b) the expression is influenced by estradiol (E2), and (c) CB1-immunoreactivity is present in the KP afferents to GnRH neurons.

These questions prompted us to investigate the site- and cell-specific expression of CB1 in female mice using multiple labeling in situ hybridization and immunofluorescent histochemical techniques. Ovariectomized and estradiol-replaced models were used in the experiments, supplemented with mice genetically altered to maximize the visualization of KP afferents to GnRH neurons.

## Materials and methods

### Animals

Adult, female CD1 mice (*n* = 20) were used for the RNAscope in situ hybridization histochemistry (ISHH), and adult, female CD1 (*n* = 10) and Kiss1-Cre-GFP mice (Kiss1^tm1.1(cre/EGFP)Steiner)^, Jackson Laboratory, stock No. 017701, *n* = 20) were used for the immunohistochemical studies. The animals were housed under controlled lighting [12:12 h light–dark cycle, lights on at 07:00 h, and temperature (22 ± 2 °C)] conditions, with access to food and water ad libitum. The CD1 mice were ovariectomized (OVX) with 3-week recovery time, and thereafter, 15 of them received a two-step subcutaneous estrogen substitution or vehicle, as described before (Bosch et al. 2013). Briefly, the animals (*n* = 30) received on the first and the second days 0.25 or 1.5 μg 17β-estradiol benzoate (EB) in 50 μl oil (*n* = 15) or 50 μl oil vehicle (*n* = 15) at Zeitgeber time (ZT) 4–5 (Table 1). The uterus weight of the OVX + Oil mice differed significantly from those treated with EB (0.049 ± 0.014 g versus 0.16 ± 0.014 g, respectively, *p* < 0.001). The Kiss1-Cre-GFP animals were all OVX and received a viral construct AAV-EF1a-DIOhChR2 (H134R)-EYFP injection [200 nl 1:2 solution of the viral stock) into the anteroventral periventricular nucleus (AVPe, Bregma AP 0.25 mm, ML 0.2 mm, DV 4.6 mm, *n* = 5)], ARC (Bregma AP −2.15 mm, ML 0.25 mm, DV 5.25 mm, *n* = 8), or posterodorsal subdivision of the medial amygdaloid nucleus (MePD Bregma AP −1.9 mm, ML 2.0 mm, DV 4.9 mm, *n* = 7) to label the KP neurons. Three weeks after the surgery, these animals were also treated with EB similarly to the above protocol.

**Table 1:** Primary and secondary antibodies used in concurrent detection of three different tissue antigens in sections of ovariectomized 17β-estradiol benzoate-treated wild-type and Kiss1-CRE-GFP mice

Detection of	GnRH-positive cells	KP-positive cells	CB1-positive cells
*In sections of wild-type CD1 mice*			
PABs	Guinea pig anti-GnRH (#1018, gift from Dr. Erik Hrabovszky, 1:50,000)	Sheep anti-KP (#053, gift from Dr. Alain Caraty, 1:1000)	Rabbit anti-CB1 (ImmunoGenes-ABS Zrt, 1:1000)
SABs	FITC-donkey anti-guinea pig IgG (H + L) (#706-095-148, Jackson Immuno Research Laboratories, 1:500)	CY3-donkey anti-sheep IgG (Fab) 2 (#713-165-147, Jackson Immuno Research Laboratories, 1:3000)	CY5-donkey anti-rabbit IgG (H + L), (#711-175-152, Jackson Immuno Research Laboratories, 1:2000)
*In sections of Kiss1-Cre-GFP mice injected with AAV-EF1A-DIO-hChR2 (H134R)-EYFP*			
PABs	Guinea pig anti-GnRH (#1018, gift from Dr. Erik Hrabovszky, 1:50,000)	Rabbit anti-GFP, (AB10145, Millipore, 1:2000)	Goat anti-CB1 (gift from Dr. Masahiko Watanabe, 1:600)
SABs	CY5- conjugated donkey anti-guinea pig IgG (#706-175-148, Jackson Immuno Research Laboratories, 1:2000)	FITC-conjugated donkey anti-rabbit IgG (#711-095-152, Jackson Immuno Research Laboratories, 1:1000)	CY3-conjugated donkey anti-goat IgG (#706-165-147, Jackson Immuno Research Laboratories, 1:2000)

### RNAscope ISHH to detect KP, CB1, vesicular GABA transporter (VGAT), or vesicular glutamate transporter (VGLUT) 2 mRNAs

#### Tissue preparation for in situ hybridization

Four hours after the second EB or oil vehicle injections, the animals were sacrificed, and the brains were removed and frozen on dry ice. 14 µm thick coronal sections were cut on a Leica CM 3050 S cryostat (Leica Microsystems, Vienna, Austria), and mounted consecutively onto groups of ten Super Frost Ultra Plus glass slides (Thermo Fisher Scientific, Budapest, Hungary). Two coronal sections of OVX + EB mice (*n* = 5) 126 µm apart from each other were paired with two corresponding sections of OVX + oil mice (*n* = 5) on each slide. The slides were stored at −80 °C until processed.

### RNAScope in situ hybridization

Five slides of the rostral periventricular area of the third ventricle (RP3V) [containing the AvPe and/or the periventricular hypothalamic nucleus (Pe)], and five slides of the ARC regions (altogether 10 sections of five OVX-EB and 10 sections of five OVX + oil mice for each regions) were selected for each hybridization. The pre-treatment (#3,20,513-USM) and hybridization (#3,20,293) protocols of the RNAscope technique (Advanced Cell Diagnostics Inc., Newark, CA, USA) were used, which allowed the application of the Multiplex reagent kit. The hybridization cocktail contained the following probes: for channel 1, it was Kiss1 (catalog #476291_O1, XM_006529679, region 121 – 1376); for channel 2, it was either SLC32A1 (VGAT, catalog #3,19,191-C2, NM_009508.2, region 894–2037) or SLC17A6 (VGLUT2; catalog #3,19,171-C2, NM_080853.3, region 1986–2998); and for channel 3, it was CNR1(CB1, catalog # 4,20,721-C3, NM_007726.3, region 530–1458). The “B” variant of Amp4 was used, which labeled channel 1–3 with Alexa488 (green), Atto550 (red), and Atto647 (far red), respectively. The sections were coverslipped with Prolong Antifade kit (Molecular Probes, Leiden, The Netherlands) after the amplification/labeling steps and counterstaining with DAPI.

### Capturing and analyzing the RNAScope signals

The quadruple-labeled sections were scanned in a Nikon C2 confocal microscope (Nikon, Japan) using the 20 × objective. Multiple stacks of optical slices (1024 × 1024 pixels, z-steps 0.6 µm) were obtained from the −0.6 to −2.4 µm layer of the sections by scanning the full RP3V and ARC regions on one side. The fluorochromes were excited with laser lines 488, 561, and 641 nm. The DAPI nuclear staining was also detected using the 405 nm laser. Laser intensities and other acquisition parameters were kept the same during the whole scanning. Using the Image J software, the different channels in the image stack were combined into maximum intensity projections, and binarized and merged into single TIFF images. The DAPI channel was also saved as separate TIFF images. Lit pixels were counted within regional and cellular borders, which were determined manually based on the DAPI image and kisspeptin signals. A cell was deemed as positive for a given mRNA when the number of lit pixels within the cell area were higher (containing more than five pixels) than the number of lit pixels in identical sized area of the white matter (maximum five pixels). Data were analyzed by one-way ANOVA, and significant difference was determined by the Holm–Sidak method.

### Tissue preparation for detecting the viral fluorescent tracer and immunofluorescence

The animals were perfused transcardially with phosphate-buffered saline (PBS 0.1 M) containing 4% paraformaldehyde (PFA). The brains were removed, post fixed for 24 h, and transferred into 30% sucrose for cryoprotection, and then, 30 µm thick coronal sections were cut on a freezing microtome by collecting every third sections into the same well.

### Evaluation of viral tracing of KP fibers

A group of sections were mounted from each brain with RP3V (*n* = 5), ARC (*n* = 8), or MEA (*n* = 7) injections of the viral tracer. Brains showing successful labeling of KP neurons in consecutive sections were selected for subsequent multiple-label immunofluorescence staining of YFP in KP cells, GnRH, and CB1.

### Triple-label immunofluorescence

After the endogenous peroxidase activity had been quenched with 0.5% hydrogen peroxide (20 min), sections were permeabilized with 0.5% Triton X-100 (23,472–9, Sigma, 20 min), and treated with 2% normal horse serum (20 min) to reduce non-specific antibody binding. All treatments and interim rinses in PBS (3 × 5 min) were carried out at room temperature, except for incubation in the primary antibodies and fluorochromes, which was carried out at 4 °C. Sections were incubated in a cocktail of the primary antibodies for 72 h and the fluorochrome-labeled secondary antibodies overnight with a 2 h rinse in TRIS at RT in between. Primary and secondary antibodies used in the immunohistochemical procedure are listed in Table 1.

Thus, guinea pig anti-GnRH (#1018, gift from Dr. Erik Hrabovszky, 1:50,000) (Hrabovszky et al. 2011), sheep anti-KP (#053, gift from Dr. Alain Caraty, 1:1000) and rabbit anti-CB1, ImmunoGenes-ABS Zrt, 1:1000) primary antibodies, and FITC-donkey-anti-guinea pig IgG (H + L) (#706-095-148, Jackson ImmunoResearch Laboratories, 1:500), CY3-donkey-anti-sheep IgG (Fab)2 (#713-165-147, Jackson ImmunoResearch Laboratories, 1:3000), and CY5-donkey-anti-rabbit IgG (H + L), 711-175-152, Jackson ImmunoResearch Laboratories, 1:2000) secondary antibodies were used to stain sections of CD1 mice. To maximize the visualization of the axonal boundaries of KP neurons, mice expressing YFP in KP cells of the virus injected KP-CRE animals were used for the immunofluorescent detection of CB1 in afferents of GnRH neurons. In this case, guinea pig anti-GnRH (#1018, 1:50,000) (Hrabovszky et al. 2011), rabbit anti-GFP (for KP, AB10145 Millipore, 1:2000), and goat anti-CB1, 1:600 (Makara et al. 2007), primary antibodies, and CY5-conjugated donkey anti-guinea pig IgG (#706-175-148, Jackson ImmunoResearch Laboratories, 1:2000, 2 h), FITC-conjugated donkey anti-rabbit IgG (#711-095-152, Jackson ImmunoResearch Laboratories, 1:1000, 2 h), and CY3-conjugated donkey anti-goat IgG (#706-165-147, Jackson ImmunoResearch Laboratories, 1:2000, 2 h) secondary antibodies were used. Sections were then rinsed in TRIS (2 h), mounted onto glass slides and cover slipped with Moviol.

### Confocal microscopy and 3-D reconstruction of KP afferents to GnRH-IR cells

The triple-labeled sections were scanned in a Nikon A1R confocal microscope (Nikon, Japan) using the  × 20 and the 60 × oil immersion objectives. Multiple stacks of optical slices (1024 × 1024 pixels, z-steps 0.15 µm) were generated, which contained the KP-IR fibers in apposition to the cell bodies and the processes of GnRH-IR neurons. The separately recorded green, red, and far-red channels were merged and displayed with the ImageJ software running on an IBM compatible personal computer. Orthogonal views from different planes (*x*/*y*, *x*/*z* or *y*/*z*) of the confocal microscopic images were used to analyze the KP-immunoreactive fibers for apposition to GnRH neurons and CB1-immunoreactivity. To enable three-dimensional (3D) analyses, the images were further processed using the software Amira (6.0, Visual Imaging Group). The stacks of the optical slices were loaded into the visualization program and rendered in three dimensions with surfaces generated from above threshold immunoreactivity. The threshold was set individually for each image and color channel to minimize any noise, while maintaining the proper cellular boundaries. The surfaces generated from the three channels in the same optical volume were visualized to check for cell-to-cell contacts, and the presence of CB1-immunoreactivity in KP fibers associated with GnRH neurons. This enabled verification of the findings from the two-dimensional confocal image analyses.

GnRH neurons (*n* = 6 per brains with successful viral labeling of RP3V kisspeptin neurons) located in the medial preoptic area and seen to be a candidate of receiving KP-IR afferents at 20 × magnifications were selected for 60 × scans and analyses. An axon was positive for CB1 when immunoreactivity was detected inside or in association with the cell membrane of the YFP-positive KP processes. No such signal was detected in CB1-KO mice (kindly provided by A. Zimmer, University of Bonn and bred at the Medical Gene Technology Unit of the Institute of Experimental Medicine).

## Results

### Expression of KP, CB1, VGLUT2, and VGAT in the RP3V and ARC regions: effect of estrogen

**Fig. 1:**
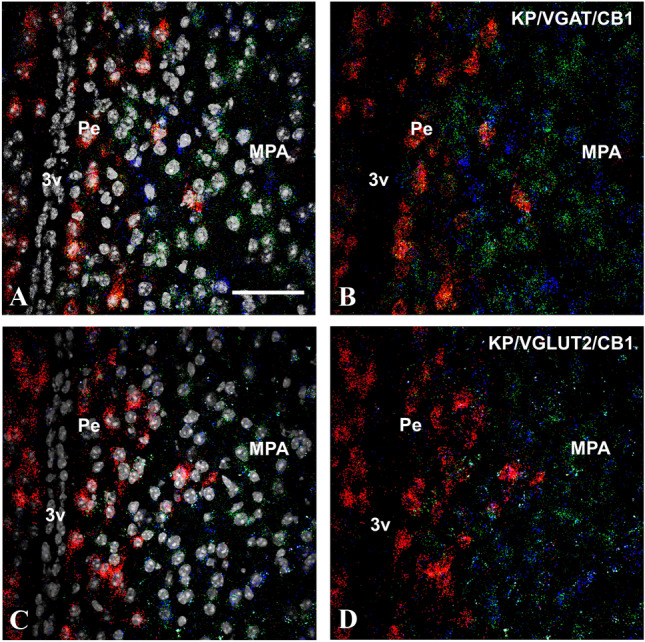
Kisspeptin (KP, red), type 1 cannabinoid receptor (CB1, blue) and vesicular GABA (VGAT, green) or glutamate transporter (VGLUT2, green) transcripts detected by the RNAscope in situ hybridization technique in the preoptic area of OVX + EB mice. DAPI counterstaining (grey–white spots) demonstrates the cell nuclei and supports the area and cell-specific identification of the different transcripts (**A** and **C**). The KP mRNA signal is predominantly present in the periventricular region of the preoptic area, whereas the other transcripts appear also abundantly in the medial preoptic area (**B** and **D**). Scale bar 50 µm

**Fig. 2:**
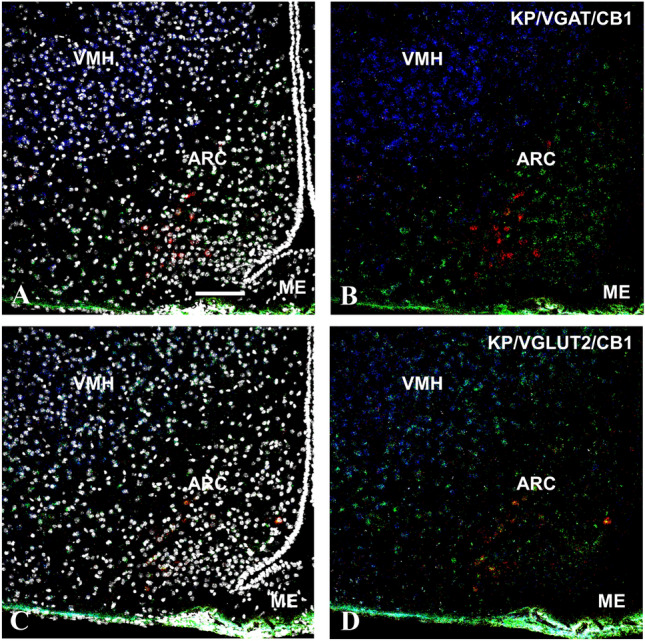
Kisspeptin (KP, red), type 1 cannabinoid receptor (CB1, blue), and vesicular GABA (VGAT, green) or glutamate transporter (VGLUT2, green) transcripts detected by the RNAscope in situ hybridization technique in the medial basal hypothalamus of OVX + Oil mice. DAPI counterstaining (grey–white spots) demonstrates the cell nuclei and supports the area and cell-specific identification of the different transcripts (**A** and **C**). The KP mRNA signal is present in the arcuate nucleus, where a low level of CB1 transcript can also be detected (**B** and **D**). In contrast, the VMH shows a much stronger CB1 mRNA signal in co-distribution almost exclusively with the VGULT2 mRNA signal (**B** and **D**). Scale bar 100 µm

**Fig. 3:**
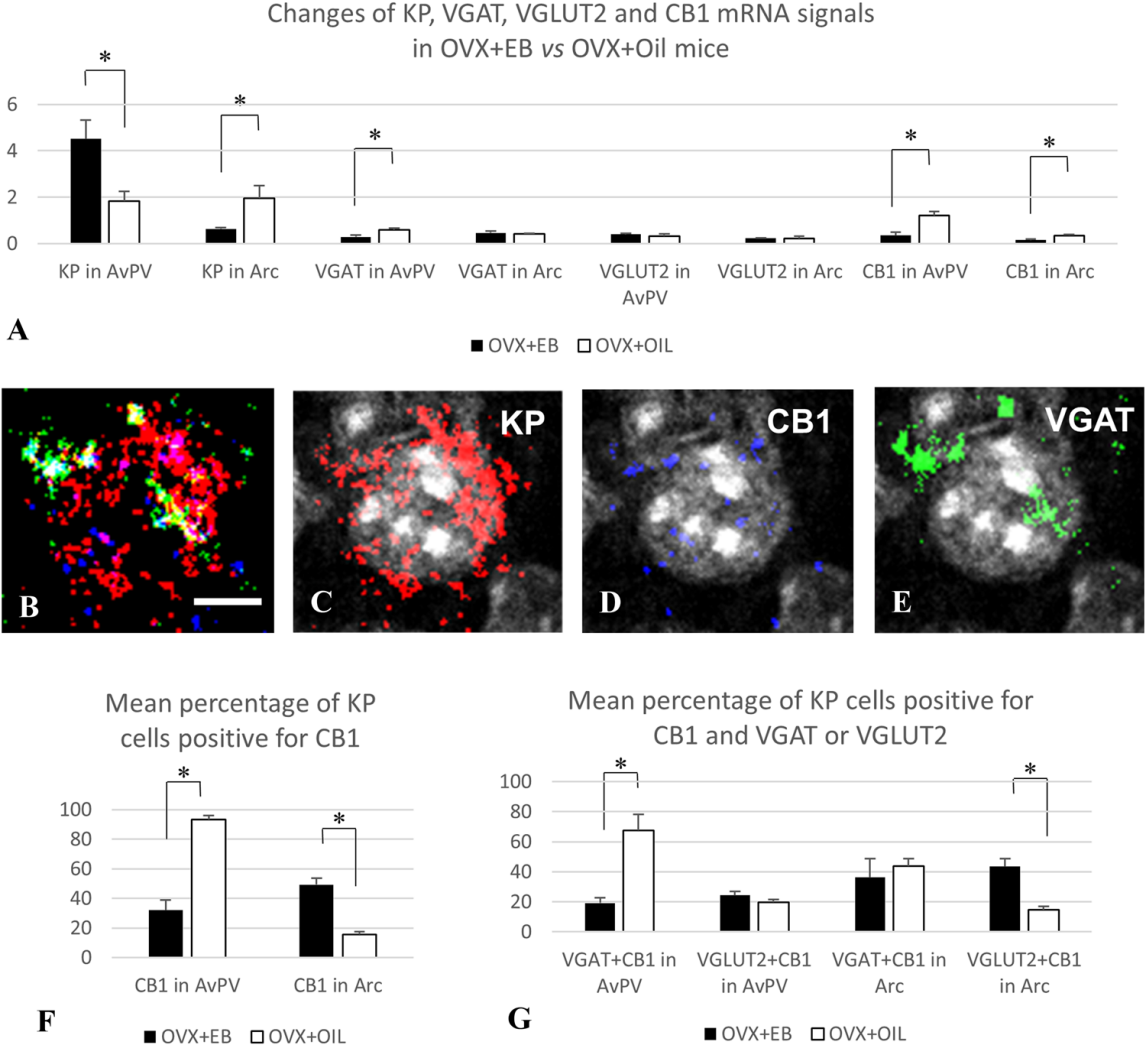
RNAscope in situ hybridization signals detected at area (**A**) and cellular (**B**–**G**) levels in the RP3V and Arc of OVX mice treated with EB or oil vehicle. Co-distribution of signals for KP (red), CB1 (blue), and VGAT (green) in association with a preoptic cell nucleus (grey–white) (**B**–**E**). Mean level of signals (**A**, determined by the number of positive pixels/ROIs in the RP3V and Arc). Expression of CB1 mRNA in KP neurons (**F**, determined by the presence of pixels identifying CB1 in KP mRNA-positive cells) and co-localization of CB1 mRNA signal with VGAT or VGLUT2 mRNA signals in KP mRNA-positive cells of the RP3V and Arc (**G** determined by the presence of pixels identifying CB1 and VGAT or VGLUT2 mRNA signals in KP mRNA-positive cells). Scale bar in **B**–**E** 5 µm *p* < 0.05, significant difference labeled with asterisk

The RNAscope in situ hybridization technique detected mRNA signals for KP, CB1, VGLUT2, and VGAT concurrently in ovariectomized mouse models treated for 2 days either with EB or oil vehicle. In agreement with the previous reports (Gottsch et al. 2009), the KP mRNA signal showed an estrogen-dependent alteration in the RP3V (Fig. 1) and ARC (Fig. 2), and this has validated the animal models used in this study. Thus, there was a profound and area-specific effect of estrogen-treatment on the level of KP mRNAs in the RP3V and ARC (Fig. 3A). While EB-treatment significantly increased the mRNA levels for KP in the RPRV region (from 1.83 ± 0.41 to 4.51 ± 0.8, *p* < 0.02), the same treatment reduced it in the ARC (from 1.95 ± 0.56 to 0.63 ± 0.05, *p* = 0.02). The expression levels of VGAT and VGLUT2 were relatively low in both regions compared to the neighboring medial preoptic area (MPA) and/or the ventromedial hypothalamic nucleus (VMH) (Figs. 1 and 2). No significant differences were detected between the two animal models for VGAT mRNAs in the ARC and VGLUT2 mRNAs in both the RPRV and ARC. The VGAT mRNA signal was, however, significantly lower (*p* = 0.028) in the RP3V region of OVX-EB animals (0.29 ± 0.26) than in the OVX + OIL group (0.59 ± 0.07) (Fig. 3A). The CB1 mRNA was also relatively low in the RP3V and ARC compared to the neighboring MPA and VMH regions (Figs. 1 and 2), respectively. Additionally, CB1 mRNA showed an estrogen-sensitivity with signal being lower both in the RP3V (0.35 ± 0.14 vs 1.2 ± 0.19, *p* = 0.02) and ARC (0.33 ± 0.05 vs 0.16 ± 0.04, *p* = 0.05) of EB-treated mice compared to mice receiving vehicle only (Fig. 3A).

### Expression of CB1 in RP3V and Arc KP neurons: effect of estrogen

The same sections were analyzed at cellular levels in the RP3V and ARC for co-expression of KP with CB1 and either VGAT or VGLUT2 (Fig. 3B–E). In the RP3V, 93.4 ± 2.7% of KP neurons expressed CB1 in the OVX + OIL group, and 32 ± 6.9% of them in the OVX + EB group, whereas in the ARC, these values were 15.5 ± 2 and 49.3 ± 4.4%, respectively (Fig. 3F).

### Expression of CB1 in GABAergic and glutamatergic subpopulations of KP neurons

KP neurons express VGAT and VGLUT2 mRNAs both in the RP3V and the Arc. In the RP3V, 48.4 ± 10.6% of KP neurons expressed VGAT in the OVX + OIL group, and 69.2 ± 13.4% of them in the OVX + EB group, whereas in the ARC, these values were 76.5 ± 2.8 and 73.5 ± 5.9%, respectively. Concerning the co-expression of VGLUT2 with KP, the value was 23.5 ± 1.9% in the RP3V of OVX + OIL group, and 31.4 ± 2.5% in the same region of the OVX + EB group, whereas these values were in the ARC 83.7 ± 2.4 and 88.3 ± 3.8%, respectively. The estrogen level of the animals had no significant influence on the percentage of KP neurons co-expressing VGAT or VGLUT. The percentage of VGLUT2-positive KP neurons was, however, significantly higher in the ARC than in the RP3V (*p* < 0.001) (data not shown). When CB1 mRNA expression was analyzed in the VGAT- or VGLUT2-positive KP neurons, a profound site-specific effect of EB was revealed in the RP3V and ARC. Thus, the percentage of the CB1 expressing VGLUT2-positive KP neurons was significantly increased in the ARC of estrogen-treated OVX mice, compared to the oil-treated animals (43.5 ± 5 vs. 14.8 ± 2.4%, *p* < 0.005). The same treatment resulted in a significant decrease of CB1 expression in VGAT-positive KP neurons in the RP3V region of the brain (19.1 ± 3.5 vs. 67.3 ± 10.9%, *p* < 0.05) (Fig. 3F).

### CB1-immunoreactivity in KP afferents to GnRH neurons

**Fig. 4:**
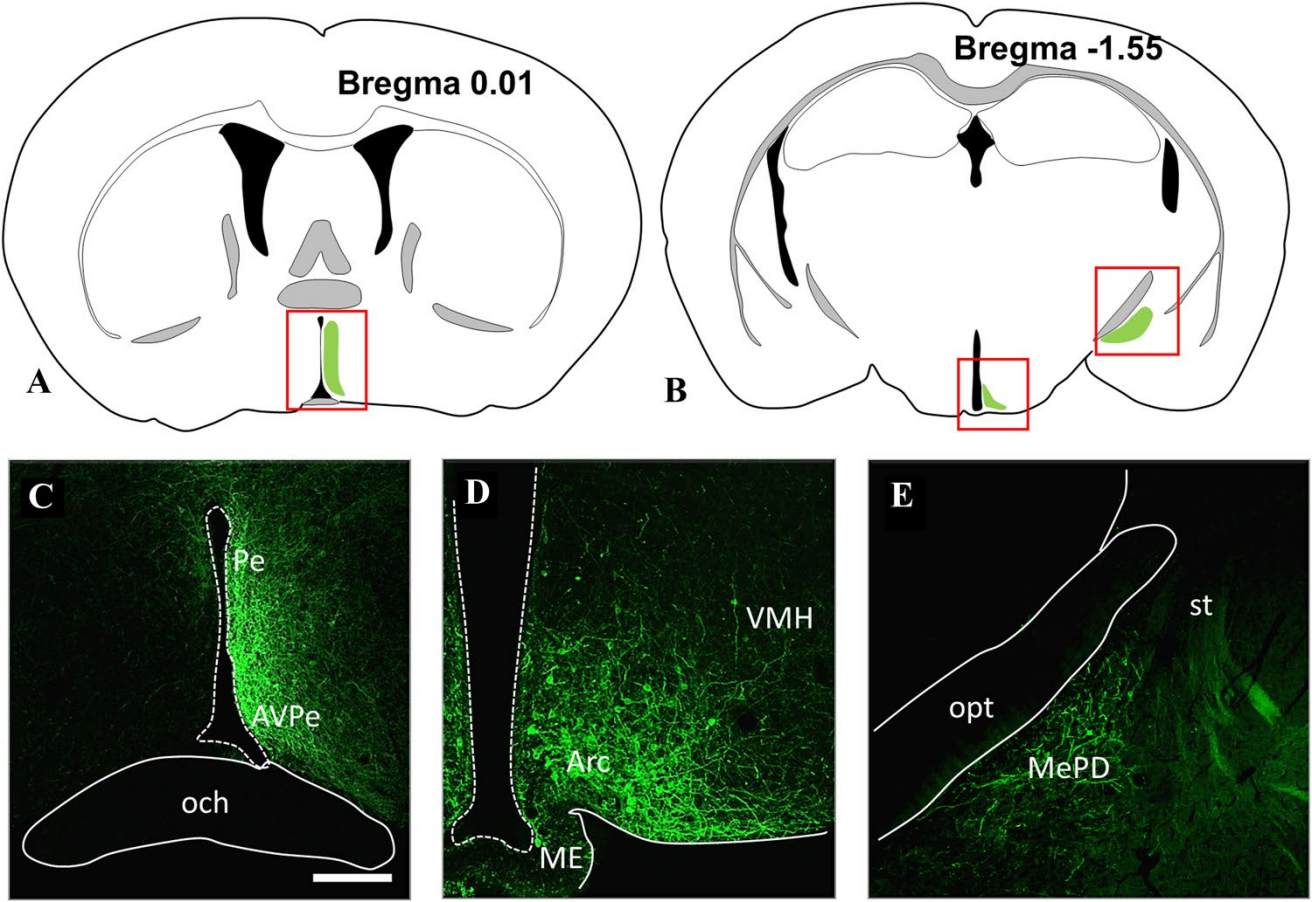
Illustration of the brain areas containing Cre expressing KP neurons (**A** and **B**), which underwent a virus-based identification in the preoptic (**C**), arcuate (**D**), and medial amygdala (**E**) regions, respectively. The expressed YFP has been immunohistochemically amplified to show membranes of KP neurons. Modifications of the atlas images from the mouse brain atlas of Paxinos and Franklin’s (2012). Scale bar 200 µm. *Arc* arcuate nucleus, *AVPe* anteroventral periventricular nucleus, *ME* median eminence, *MePD* posterodorsal subdivision of medial amygdala, *och* optic chiasm, *opt* optic tract, *Pe* periventricular hypothalamic nucleus, *st* stria terminalis, VMH ventromedial hypothalamic nucleus

**Fig. 5:**
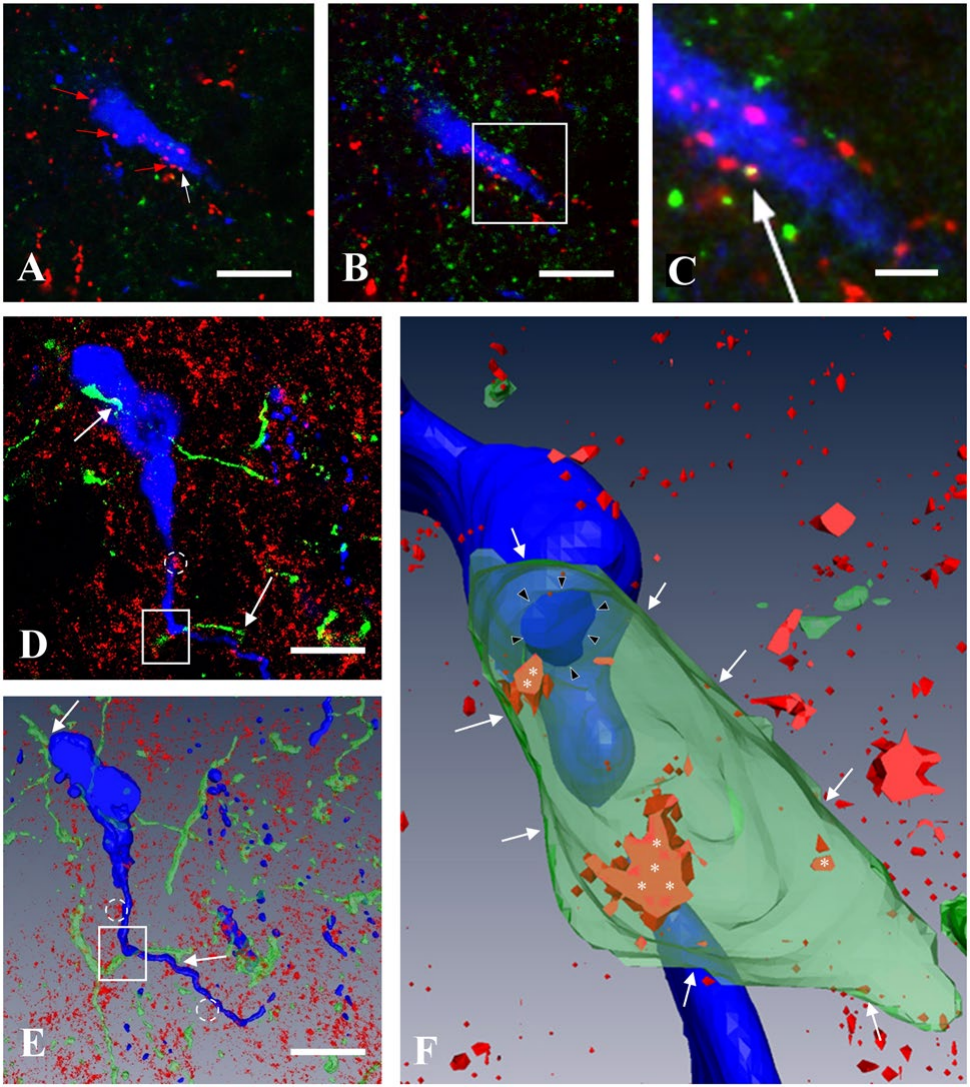
CB1-immunoreactivity in KP afferents of GnRH neurons in the medial preoptic area of OVX-EB mice. Demonstration of appearance of CB1-immunoreactivity in one of the KP-IR afferent fiber (white arrow) in confocal microscopic Z-stack series. **A** A single optical slice shows multiple KP-immunoreactive (IR) varicosities (red and white arrows) in apposition to a GnRH-IR neuron (blue). **B** is an adjacent optical slice. The boxed area in this image is magnified in **C** to demonstrate the yellow-colored double-labeled KP-IR varicosity (white arrow). **D** Yellow fluorescent protein (YFP)-positive, green-colored axon varicosities (KP fibers after viral and immunohistochemical detection) in apposition to two adjacent gonadotropin-releasing hormone (GnRH)-IR cells (blue) shown in merged three optical slices. **E** The 3D rendered view of all optical slices of the same structures. The GnRH neurons are embedded in a tissue showing punctate CB1-IR sites (red), where CB1-IR clusters (dotted circles) and KP-IR fibers (white arrows) are in association with GnRH-IR cell bodies or processes. **F** The projection image of the 3D reconstructed area of **E** (white rectangle) is shown at higher power. Some of the CB1-IR sites (white asterisks) are visible only, if the KP-IR fiber is made semi-transparent. The CB1-IR sites in the membrane of the KP-IR fiber (white arrows) turn up in orange-red color marking co-localization. The contact site between the KP fiber and the GnRH dendron is marked by black arrowheads. Scale bar 10 µm (on **A**, **B**, **D**, **E**) and 5 µm (on **C**)

The presence of CB1 in KP afferents of GnRH neurons was studied in CD1 and KP-CRE mice (Fig. 4) by immunohistochemical labeling of preoptic sections for CB1, GnRH, and KP (Fig. 5A–C) or alternatively YFP (Fig. 5D–F), expressed after transmission of its gene by viral-infection of KP-CRE neurons. Because of its dominant membrane localization, CB1 appeared rarely in overlap with KP-immunoreactivity marking primarily secretory granules in the axon terminals (Fig. 5A–C). Therefore, axonal projections of KP-CRE-expressing neurons were traced with yellow fluorescent proteins, which also marked the borders of axon terminals in contact with GnRH neurons (Fig. 5D–F).

The viral construct (AAV-EF1a-DIOhChR2 (H134R)-EYFP) was delivered to all major KP-populations of the mouse brain, where it was translated to EYFP and transported together with the ChR2 to the processes of KP-CRE cells including the cell membrane (Fig. 4). YFP-positive processes in apposition to GnRH neurons were rarely seen in the POA of mice, if the viral construct was injected into the ARC or the MePD. In contrast, such afferents were seen more often in mice, which were targeted with the viruses in the RP3V region.

The YFP expressed by AAV-infected RP3V neurons identified the cellular borders of KP neuronal processes in the MPA, including those, which were in juxtaposition to GnRH-IR perikarya and processes (Figs. 5D–F). CB1-IR was found in about two-thirds of these KP neuronal processes (Table 2, 70 ± 2.9 and 59 ± 4.1% of them (*n* = 187) were in apposition to GnRH perikarya and processes, respectively).

**Table 2:** Presence of CB1-immunoreactivity in YFP-labeled KP afferents of GnRH neurons in four Kiss1-Cre-GFP animals, which received the viral construct AAV-EF1a-DIOhChR2 (H134R)-EYFP injection into the anteroventral periventricular nucleus

Brain#	GnRH cells	Number of appositions on the soma	Number/percentage of appositions with CB1 on soma	Number of appositions on the dendrites	Number/percentage of appositions with CB1 on dendrites
I. (#3116)	6	8	6/75%	29	19/65.5%
II. (#3143)	6	8	6/75%	35	18/51.4%
III. (#3155)	6	11	7/63.6%	47	27/57.4%
IV. (#3156)	6	9	6/66.6%	40	25/62.5%
SUM	24	36	25/69.4%	151	85/56.3%
Mean (%)			70		59
SEM			2.9		4.1

Regions containing six GnRH neurons contacted by KP fibers were selected for analysis

## Discussion

This study provides evidence that (a) CB1 mRNA is expressed by both GABAergic and glutamatergic subpopulation of kisspeptin neurons, (b) the receptor protein is present in KP afferents of GnRH neurons, and (c) the expression of CB1 mRNA shows estrogen-dependent regulation. The applied estrogen-treatment, known to induce proestrus in mice, reduced the level of CB1 transcripts in the RP3V and ARC, and differently influenced its co-localization with VGAT or VGLUT2 in kisspeptin neurons.

### Experimental model to study whether CB1 is involved in presynaptic regulation of kisspeptin afferents

Levels of CB1 expression are highly variable among different brain locations and cell types. While the cerebral cortex and hippocampus contain a very high level of CB1 protein, expression levels are relatively low in hypothalamic regions (Wittmann et al. 2007). Despite its low levels, a large body of evidence supports strong cannabinoid-dependent signaling in the regulation of hypothalamic functions (Gammon et al. 2005; Pagotto et al. 2006; Tasker 2006; Brents 2016). Concerning the reported role of CB1 in the regulation of reproduction, and the critical functions of kisspeptin neurons in this regard, in the current study, the KP cell-specific expression of CB1 was investigated using multiple labeling in situ hybridization and immunofluorescent histochemical techniques. During the ovarian cycle, the hypothalamic regulatory circuits operate under the influence of estrogens’ negative and positive feedback effects. Therefore, we have used the animal models with reduced serum ovarian hormone levels achieved by 3-week time gonadectomy (Baumgartner et al. 2019), and the hormone-replaced pairs treated subsequently with a priming and a surge-inducing dose of estrogen (Bosch et al. 2013). As expected, this treatment regime resulted in a significant difference between the uterine weights of the two models by increasing the weights fourfold compared to controls. In addition, the appearance of KP transcript levels showed high estrogen-dependency in the RP3V and ARC regions, as it was reported earlier (Lehman et al. 2010, Navarro et al. 2009, Smith et al. 2005). Thus, in the RP3V, KP transcript levels were high in the estrogen-treated animals, whereas strong signals were detected in the ARC in the absence of estrogen (Fig. 3A). The estradiol-treated model was chosen also to investigate the afferents on GnRH neurons based on the observation of Chan et al. (2011). They reported that the positive feedback levels of estradiol stimulate a robust increase in spine density and most likely in synaptic inputs of GnRH neurons.

### Distribution of CB1 in hypothalamic GABAergic and glutamatergic neurons in previous studies

Our previous mapping studies in male mice (Hrabovszky et al. 2012) revealed a differential expression of mRNAs for CB1, the GABAergic marker GAD65, and the glutamatergic marker VGLUT2 in the RP3V and ARC regions, where the two major subpopulations of KP neurons are located. Relatively low abundance of the hybridization signal and high-to-moderate-to-low number of labeled cells were found for CB1 in the AVPe, the Pe, and the ARC nucleus, respectively. CB1 mRNA was detected in both GABAegic and glutamatergic neurons in these regions. In the current study, the mRNA signals for CB1, VGAT and VGLUT2 were relatively low in both the RP3V and ARC of female mice and their relative abundance compared to the neighboring brain areas (i.e., MPA or the VMH) was similar to the one detected previously in male mice (Hrabovszky et al. 2012).

### Estrogenic regulation of hypothalamic CB1, VGAT, and VGLUT2 expression

Estrogen treatment resulted in a down-regulation of CB1 expression in both regions (Fig. 3A). This agrees with the observation of Riebe and Gorzalka, (Riebe et al. 2010), showing that OVX females have higher amounts of hypothalamic cannabinoid receptor binding relative to both cycling and OVX + E2 females. The current result, therefore, might indicate a reduced involvement of CB1 mediating suppression in local circuit activity, which generate higher frequency postsynaptic events in target cells in the presence of estrogen (Glanowska and Moenter 2011).

Similarly to CB1, expression of VGAT mRNA was lower in RP3V of the OVX + EB mice compared to the oil-treated ones. This seems to be congruent with the observation of Ottem et al. (Ottem et al. 2004) showing a decrease of the VGAT-containing vesicles in the RP3V of female rats at the time of the surge.

Gonadal hormones were reported to alter also the VGLUT2 expression in these regions. E2-treatment was shown to increase the VGLUT2 immunoreactive vesicles in the afferents of GnRH neurons in ovariectomized rats (Ottem et al. 2004). An increase of VGLUT2 mRNAs by estrogen-treatment was reported also in the ARC of ovariectomized mice (Qiu et al. 2018). The positive effect of gonadal hormones on VGLUT2 expression is, however, not unambiguous, since gonadectomy led to a significant elevation of VGLUT2 mRNA in male mice, compared to the levels observed in gonad intact conditions (Nestor et al. 2016).

The comparison of current RNAscope signals of the EB or oil-treated animals, however, did not reveal a significant difference for VGLUT2 signals neither in the RP3V nor in the ARC. This may indicate the presence of mixed glutamatergic cell populations in these regions in which expression of VGLUT2 is differently or even antagonistically regulated by estrogen.

### Expression of CB1 in preoptic and arcuate KP neurons

Analyses of the RNAscope data at cellular levels revealed the presence of CB1 transcripts in KP neurons in both the RP3V and ARC regions, but the hormonal conditions had a great impact on the level of co-expression. Nearly all preoptic KP neurons expressed CB1 in the OVX + OIL group (93.4 ± 2.7%), but this value fall to one-third in the OVX + EB group (32 ± 6.9%). The opposite was observable for ARC KP neurons, showing low levels of co-expression in the OVX + OIL group (15.5 ± 2%), whereas CB1 mRNA expression was detectable in nearly half of the KP neurons in the OVX + EB group (49.3 ± 4.4%). By involving data on the mRNA levels for VGAT and VGLUT2 in the analyses, the presence of CB1 mRNA was also examined separately in the GABAergic and glutamatergic subpopulations of KP neurons. Both subpopulations proved to be positive for CB1 mRNA and the co-localization levels varied again according to the hormonal status of the animals. The percentage of the GABAergic KP neurons expressing CB1 was significantly lower in the RP3V of OVX + EB-treated mice, indicating that CB1 signaling might be reduced or suspended in this cell population at high estrogen levels.

Conversely, the percentage of the glutamatergic KP neurons expressing CB1 (Fig. 3F) was significantly higher in the ARC of the estrogen-treated animals compared to controls. The dramatic differences seen in the preoptic versus arcuate co-localization levels can be explained by the antagonistic regulation of KP transcript in these regions, which is combined with the reduced expression of CB1 mRNA in response to estrogen-treatment in both locations. Thus, lower co-localization levels can be found in RP3V in the OVX + EB model and in the arcuate nucleus in the OVX + Oil model, in which models the KP expression is high and the CB1 message is downregulated.

### Colocalization of KP and VGAT or VGLUT2 mRNAs

Both VGAT or VGLUT2 mRNA-containing KP cells could be detected in the preoptic and ARC regions, which is in concordance with studies detecting VGAT/or VGLUT2 mRNA in KP-CRE mice (Cravo et al. 2011) or KP-immunoreactivity in VGAT-CRE or VGLUT2-CRE mice (Cheong et al. 2015). Thus, about half to two-third of the KP neurons were positive in the current study for VGAT mRNA, and about one quarter to one-third of KP neurons were positive for VGLUT2 mRNA in the RP3V. Most of the ARC KP neurons were also positive for VGLUT2 mRNA and three quarter of them also expressed the VGAT mRNA. Of note, the level of co-localization of KP and VGAT or VGLUT mRNAs may depend on the animal model used, since quantitative PCR studies were able to find relatively few, or no RP3V KP cells with Slc17a6 (VGLUT2) mRNA or no ARC KP cells with Slc32a1 (VGAT) mRNA (Qiu et al. 2018) in Kiss1Cre:GFP mice (Gottsch et al. 2011). Furthermore, translation of neither amino acid transporter markers has been proved to functional transporter proteins, respectively, in the RP3V or ARC KP cells of these animals (Qiu et al. 2018).

### Potential role of CB1-mediated signaling in KP–GnRH interactions

KP neurons in all major locations are involved in the regulation of GnRH secretion. We have traced projections of KP neurons located in the RP3V, ARC, and MePD (Fig. 4), and in concordance with the previous observations (Kallo et al. 2012; Pineda et al. 2017; Yeo 2013; Yeo and Herbison 2011; Yeo et al. 2019; Wintermantel et al. 2006), found KP fibers in apposition to GnRH cells to originate primarily from the RP3V cells.

CB1-IR was found in the majority of these KP neuronal processes independent of being in apposition to the perikaryon or proximal processes of GnRH neurons (Fig. 5C and F). This suggests that neurotransmitter release from the KP afferents to GnRH neurons is also under regulation of endocannabinoids.

The endocannabinoid control is inhibitory by reducing the frequency of mPSCs and the firing activity of GnRH neurons in slices of metestrus female mice (Balint et al. 2016). This phase of the cycle is characterized by low serum estrogen levels, which triggers the synthesis and release of 2-AG from GnRH neurons via activating membrane-associated, rapid ERβ-mediated processes in these neurons. Acute administration of a high dose of estrogen to the slice had no effect on this inhibition, indicating that rapid, non-genomic actions of estrogen might not interfere with the CB1 signaling. The inhibition, however, does not seem to operate at high serum estrogen levels in proestrus GnRH neurons (Farkas et al. 2018). A possible explanation for this could be a longer exposure to and most likely genomic action of estrogen seen in the current study, which resulted in the down-regulation of CB1 expression in both the RP3V and ARC KP neurons. Whether it has involved both the GABAergic and glutamatergic KP afferents of GnRH neurons needs to be further investigated. However, the down-regulation of CB1 in the glutamatergic KP afferents may have contributed to the appearance of the glutamate receptor mediated PSC-es in the GnRH neurons seen on the proestrus afternoon (Farkas et al. 2018).

### Potential role of CB1-mediated signaling in KP–KP interactions

Glutamate released by ARC KP neurons targets KP neurons in this brain region and may contribute to the synchronized activity increase in populations of KP neurons during pulse generation. Glutamate released by ARC KP neurons also targets preoptic KP neurons, which in turn can enhance stimulation of GnRH neurons leading ultimately to a GnRH surge (Qiu et al. 2016). Therefore, a down-regulation of CB1 mRNA expression in the glutamatergic KP neurons by the preovulatory estrogen rise would facilitate an increased firing of KP afferents to other KP neurons, which in turn could enhance the activity of GnRH neurons.

### Potential role of CB1-mediated signaling in KP–POMC interactions

KP neurons also innervate the Arc POMC neurons. The glutamatergic and GABAergic afferents of POMC neurons are under tonic, endocannabinoid-induced inhibition (Hentges et al. 2005), which seems to be differentially regulated by estrogen. The estrogen-treatment, given 24 h prior to experimentation, increased the mEPSC frequency, and markedly decreased the potency of CB1 agonists to decrease mEPSC frequency in POMC neurons. In contrast, estrogen potentiated the cannabinoid-induced decrease in mIPSC frequency (Nguyen and Wagner 2006). The opposing effects of estrogen on the cannabinoid regulation of amino acid neurotransmission lead ultimately to the excitation of POMC neurons. This shows similarities with the effect of estrogen on the tonic endocannabinoid signaling of GnRH neurons. As the GABAergic input of GnRH neurons is facilitatory due to their high intracellular chloride levels (DeFazio et al. 2002), an estrogen-mediated down-regulation of CB1 in both GABAergic and glutamatergic afferents, including those originating from KP neurons, could potentially increase the excitation of GnRH neurons. These needs, however, to be confirmed by electrophysiological recordings.

## Conclusions

Based on the current results, estrogen seems to play a fundamental role in the regulation of CB1 expression in the different KP subpopulations of the hypothalamus. Estrogenic regulation of CB1 expression in KP cells may release neurotransmitter release from tonic endocannabinoid suppression and contribute to the modification of GABAergic and glutamatergic input of the different target cells, including the GnRH neurons.

## References

[CR1] Balint F, Liposits Z, Farkas I (2016). Estrogen receptor beta and 2-arachidonoylglycerol mediate the suppressive effects of estradiol on frequency of postsynaptic currents in gonadotropin-releasing hormone neurons of metestrous mice: an acute slice electrophysiological study. Front Cell Neurosci.

[CR2] Bardoczi Z, Wilheim T, Skrapits K, Hrabovszky E, Racz G, Matolcsy A, Liposits Z, Sliwowska JH, Dobolyi A, Kallo I (2018). GnRH neurons provide direct input to hypothalamic tyrosine hydroxylase immunoreactive neurons which is maintained during lactation. Front Endocrinol (lausanne).

[CR57] Baumgartner NE, Grissom EM, Pollard KJ, McQuillen SM, Daniel JM (2019). Neuroestrogen-Dependent Transcriptional Activity in the Brains of ERE-Luciferase Reporter Mice following Short- and Long-Term Ovariectomy. eNeuro.

[CR3] Bosch MA, Tonsfeldt KJ, Ronnekleiv OK (2013). mRNA expression of ion channels in GnRH neurons: subtype-specific regulation by 17beta-estradiol. Mol Cell Endocrinol.

[CR4] Brents LK (2016). Marijuana, the endocannabinoid system and the female reproductive system. Yale J Biol Med.

[CR61] Chan H, Prescott M, Ong Z, Herde MK, Herbison AlE, Campbell RE (2011). Dendritic spine plasticity in gonadatropin-releasing hormone (GnRH) neurons activated at the time of the preovulatory surge. Endocrinology.

[CR5] Cheong RY, Czieselsky K, Porteous R, Herbison AE (2015). Expression of ESR1 in glutamatergic and gabaergic neurons is essential for normal puberty onset, estrogen feedback, and fertility in female mice. J Neurosci.

[CR6] Clarke IJ, Arbabi L (2016). New concepts of the central control of reproduction, integrating influence of stress, metabolic state, and season. Domest Anim Endocrinol.

[CR7] Clarkson J, Herbison AE (2006). Postnatal development of kisspeptin neurons in mouse hypothalamus; sexual dimorphism and projections to gonadotropin-releasing hormone neurons. Endocrinology.

[CR8] Clarkson J, Herbison AE (2011). Dual phenotype kisspeptin-dopamine neurones of the rostral periventricular area of the third ventricle project to gonadotrophin-releasing hormone neurones. J Neuroendocrinol.

[CR9] Clarkson J, d'Anglemont de Tassigny X, Colledge WH, Caraty A, Herbison AE (2009). Distribution of kisspeptin neurones in the adult female mouse brain. J Neuroendocrinol.

[CR10] Comninos AN, Anastasovska J, Sahuri-Arisoylu M, Li X, Li S, Hu M, Jayasena CN, Ghatei MA, Bloom SR, Matthews PM, O'Byrne KT, Bell JD, Dhillo WS (2016). Kisspeptin signaling in the amygdala modulates reproductive hormone secretion. Brain Struct Funct.

[CR11] Cravo RM, Margatho LO, Osborne-Lawrence S, Donato J, Atkin S, Bookout AL, Rovinsky S, Frazao R, Lee CE, Gautron L, Zigman JM, Elias CF (2011). Characterization of Kiss1 neurons using transgenic mouse models. Neuroscience.

[CR12] DeFazio RA, Heger S, Ojeda SR, Moenter SM (2002). Activation of A-type gamma-aminobutyric acid receptors excites gonadotropin-releasing hormone neurons. Mol Endocrinol.

[CR13] Di S, Malcher-Lopes R, Halmos KC, Tasker JG (2003). Nongenomic glucocorticoid inhibition via endocannabinoid release in the hypothalamus: a fast feedback mechanism. J Neurosci.

[CR14] Farkas I, Kallo I, Deli L, Vida B, Hrabovszky E, Fekete C, Moenter SM, Watanabe M, Liposits Z (2010). Retrograde endocannabinoid signaling reduces GABAergic synaptic transmission to gonadotropin-releasing hormone neurons. Endocrinology.

[CR15] Farkas I, Balint F, Farkas E, Vastagh C, Fekete C, Liposits Z (2018). Estradiol increases glutamate and GABA neurotransmission into GnRH neurons via retrograde NO-signaling in proestrous mice during the positive estradiol feedback period. eNeuro.

[CR16] Gammon CM, Freeman GM, Xie W, Petersen SL, Wetsel WC (2005). Regulation of gonadotropin-releasing hormone secretion by cannabinoids. Endocrinology.

[CR62] Glanowska KM, Moenter SM (2011). Endocannabinoids and prostaglandins both contribute to GnRH neuron-GABAergic afferent local feedback circuits. J Neurophysiol.

[CR17] Goodman RL, Lehman MN, Smith JT, Coolen LM, de Oliveira CV, Jafarzadehshirazi MR, Pereira A, Iqbal J, Caraty A, Ciofi P, Clarke IJ (2007). Kisspeptin neurons in the arcuate nucleus of the ewe express both dynorphin A and neurokinin B. Endocrinology.

[CR18] Gottsch ML, Navarro VM, Zhao Z, Glidewell-Kenney C, Weiss J, Jameson JL, Clifton DK, Levine JE, Steiner RA (2009). Regulation of Kiss1 and dynorphin gene expression in the murine brain by classical and nonclassical estrogen receptor pathways. J Neurosci.

[CR19] Gottsch ML, Popa SM, Lawhorn JK, Qiu J, Tonsfeldt KJ, Bosch MA, Kelly MJ, Ronnekleiv OK, Sanz E, McKnight GS, Clifton DK, Palmiter RD, Steiner RA (2011). Molecular properties of Kiss1 neurons in the arcuate nucleus of the mouse. Endocrinology.

[CR20] Hentges ST, Low MJ, Williams JT (2005). Differential regulation of synaptic inputs by constitutively released endocannabinoids and exogenous cannabinoids. J Neurosci.

[CR21] Higo S, Iijima N, Ozawa H (2017). Characterisation of Kiss1r (Gpr54)-expressing neurones in the arcuate nucleus of the female rat hypothalamus. J Neuroendocrinol.

[CR22] Hrabovszky E, Molnar CS, Sipos MT, Vida B, Ciofi P, Borsay BA, Sarkadi L, Herczeg L, Bloom SR, Ghatei MA, Dhillo WS, Kallo I, Liposits Z (2011). Sexual dimorphism of kisspeptin and neurokinin B immunoreactive neurons in the infundibular nucleus of aged men and women. Front Endocrinol (lausanne).

[CR23] Hrabovszky E, Wittmann G, Kallo I, Fuzesi T, Fekete C, Liposits Z (2012). Distribution of type 1 cannabinoid receptor-expressing neurons in the septal-hypothalamic region of the mouse: colocalization with GABAergic and glutamatergic markers. J Comp Neurol.

[CR24] Kallo I, Vida B, Deli L, Molnar CS, Hrabovszky E, Caraty A, Ciofi P, Coen CW, Liposits Z (2012). Co-localisation of kisspeptin with galanin or neurokinin B in afferents to mouse GnRH neurones. J Neuroendocrinol.

[CR25] Krajewski SJ, Burke MC, Anderson MJ, McMullen NT, Rance NE (2010). Forebrain projections of arcuate neurokinin B neurons demonstrated by anterograde tract-tracing and monosodium glutamate lesions in the rat. Neuroscience.

[CR58] Lehman MN, Coolen LM, Goodman RL (2010). Minireview: kisspeptin/neurokinin B/dynorphin (KNDy) cells of the arcuate nucleus: a central node in the control of gonadotropin-releasing hormone secretion. Endocrinology.

[CR26] Lehman MN, Hileman SM, Goodman RL (2013). Neuroanatomy of the kisspeptin signaling system in mammals: comparative and developmental aspects. Adv Exp Med Biol.

[CR27] Liu X, Herbison AE (2016). Kisspeptin Regulation of neuronal activity throughout the central nervous system. Endocrinol Metab (seoul).

[CR28] Makara JK, Katona I, Nyiri G, Nemeth B, Ledent C, Watanabe M, de Vente J, Freund TF, Hajos N (2007). Involvement of nitric oxide in depolarization-induced suppression of inhibition in hippocampal pyramidal cells during activation of cholinergic receptors. J Neurosci.

[CR29] Mittelman-Smith MA, Williams H, Krajewski-Hall SJ, Lai J, Ciofi P, McMullen NT, Rance NE (2012). Arcuate kisspeptin/neurokinin B/dynorphin (KNDy) neurons mediate the estrogen suppression of gonadotropin secretion and body weight. Endocrinology.

[CR30] Murakawa H, Iwata K, Takeshita T, Ozawa H (2016). Immunoelectron microscopic observation of the subcellular localization of kisspeptin, neurokinin B and dynorphin A in KNDy neurons in the arcuate nucleus of the female rat. Neurosci Lett.

[CR59] Navarro VM, Gottsch ML, Chavkin C, Okamura H, Clifton DK, Steiner RA (2009). Regulation of gonadotropin-releasing hormone secretion by kisspeptin/dynorphin/neurokinin B neurons in the arcuate nucleus of the mouse. J Neurosci.

[CR31] Nestor CC, Qiu J, Padilla SL, Zhang C, Bosch MA, Fan W, Aicher SA, Palmiter RD, Ronnekleiv OK, Kelly MJ (2016). Optogenetic stimulation of arcuate nucleus kiss1 neurons reveals a steroid-dependent glutamatergic input to POMC and AgRP neurons in male mice. Mol Endocrinol.

[CR32] Nguyen QH, Wagner EJ (2006). Estrogen differentially modulates the cannabinoid- induced presynaptic inhibition of amino acid neurotransmission in proopiomelanocortin neurons of the arcuate nucleus. Neuroendocrinology.

[CR33] Ohkura S, Uenoyama Y, Yamada S, Homma T, Takase K, Inoue N, Maeda K, Tsukamura H (2009). Physiological role of metastin/kisspeptin in regulating gonadotropin-releasing hormone (GnRH) secretion in female rats. Peptides.

[CR34] Ottem EN, Godwin JG, Krishnan S, Petersen SL (2004). Dual-phenotype GABA/glutamate neurons in adult preoptic area: sexual dimorphism and function. J Neurosci.

[CR35] Pagotto U, Marsicano G, Cota D, Lutz B, Pasquali R (2006). The emerging role of the endocannabinoid system in endocrine regulation and energy balance. Endocr Rev.

[CR36] Pineda R, Plaisier F, Millar RP, Ludwig M (2017). Amygdala kisspeptin neurons: putative mediators of olfactory control of the gonadotropic axis. Neuroendocrinology.

[CR37] Porteous R, Petersen SL, Yeo SH, Bhattarai JP, Ciofi P, de Tassigny XD, Colledge WH, Caraty A, Herbison AE (2011). Kisspeptin neurons co-express met-enkephalin and galanin in the rostral periventricular region of the female mouse hypothalamus. J Comp Neurol.

[CR38] Qiu J, Nestor CC, Zhang C, Padilla SL, Palmiter RD, Kelly MJ, Ronnekleiv OK (2016). High-frequency stimulation-induced peptide release synchronizes arcuate kisspeptin neurons and excites GnRH neurons. Elife.

[CR39] Qiu J, Rivera HM, Bosch MA, Padilla SL, Stincic TL, Palmiter RD, Kelly MJ, Ronnekleiv OK (2018). Estrogenic-dependent glutamatergic neurotransmission from kisspeptin neurons governs feeding circuits in females. Elife.

[CR40] Riebe CJ, Hill MN, Lee TT, Hillard CJ, Gorzalka BB (2010). Estrogenic regulation of limbic cannabinoid receptor binding. Psychoneuroendocrinology.

[CR41] Sawai N, Iijima N, Takumi K, Matsumoto K, Ozawa H (2012). Immunofluorescent histochemical and ultrastructural studies on the innervation of kisspeptin/neurokinin B neurons to tuberoinfundibular dopaminergic neurons in the arcuate nucleus of rats. Neurosci Res.

[CR42] Scott V, Brown CH (2013). Beyond the GnRH axis: kisspeptin regulation of the oxytocin system in pregnancy and lactation. Adv Exp Med Biol.

[CR43] Seymour AJ, Scott V, Augustine RA, Bouwer GT, Campbell RE, Brown CH (2017). Development of an excitatory kisspeptin projection to the oxytocin system in late pregnancy. J Physiol.

[CR44] Skrapits K, Borsay BA, Herczeg L, Ciofi P, Liposits Z, Hrabovszky E (2015). Neuropeptide co-expression in hypothalamic kisspeptin neurons of laboratory animals and the human. Front Neurosci.

[CR60] Smith JT, Cunningham MJ, Rissman EF, Clifton DK, Steiner RA (2005). Regulation of Kiss1 gene expression in the brain of the female mouse. Endocrinology.

[CR45] Steindel F, Lerner R, Haring M, Ruehle S, Marsicano G, Lutz B, Monory K (2013). Neuron-type specific cannabinoid-mediated G protein signalling in mouse hippocampus. J Neurochem.

[CR46] Stephens SBZ, Kauffman AS (2017). Regulation and possible functions of kisspeptin in the medial amygdala. Front Endocrinol (lausanne).

[CR47] Tasker JG (2006). Rapid glucocorticoid actions in the hypothalamus as a mechanism of homeostatic integration. Obesity (silver Spring).

[CR48] Vida B, Deli L, Hrabovszky E, Kalamatianos T, Caraty A, Coen CW, Liposits Z, Kallo I (2010). Evidence for suprachiasmatic vasopressin neurones innervating kisspeptin neurones in the rostral periventricular area of the mouse brain: regulation by oestrogen. J Neuroendocrinol.

[CR49] Wahab F, Atika B, Shahab M (2013). Kisspeptin as a link between metabolism and reproduction: evidences from rodent and primate studies. Metabolism.

[CR50] Williams WP, Jarjisian SG, Mikkelsen JD, Kriegsfeld LJ (2011). Circadian control of kisspeptin and a gated GnRH response mediate the preovulatory luteinizing hormone surge. Endocrinology.

[CR51] Wintermantel TM, Campbell RE, Porteous R, Bock D, Grone HJ, Todman MG, Korach KS, Greiner E, Perez CA, Schutz G, Herbison AE (2006). Definition of estrogen receptor pathway critical for estrogen positive feedback to gonadotropin-releasing hormone neurons and fertility. Neuron.

[CR56] Wittmann G, Deli L, Kalló I, Hrabovszky E, Watanabe M, Liposits Z, Fekete C (2007). Distribution of type 1 cannabinoid receptor (CB1)-immunoreactive axons in the mouse hypothalamus. J Comp Neurol.

[CR52] Yeo SH (2013). Neuronal circuits in the hypothalamus controlling gonadotrophin-releasing hormone release: the neuroanatomical projections of kisspeptin neurons. Exp Physiol.

[CR53] Yeo SH, Herbison AE (2011). Projections of arcuate nucleus and rostral periventricular kisspeptin neurons in the adult female mouse brain. Endocrinology.

[CR54] Yeo SH, Kyle V, Morris PG, Jackman S, Sinnett-Smith LC, Schacker M, Chen C, Colledge WH (2016). Visualisation of Kiss1 neurone distribution using a Kiss1-CRE transgenic mouse. J Neuroendocrinol.

[CR55] Yeo SH, Kyle V, Blouet C, Jones S, Colledge WH (2019). Mapping neuronal inputs to Kiss1 neurons in the arcuate nucleus of the mouse. PLoS ONE.

